# Asymmetric reconstruction of the aquareovirus core at near-atomic resolution and mechanism of transcription initiation

**DOI:** 10.1093/procel/pwad002

**Published:** 2023-02-04

**Authors:** Alexander Stevens, Yanxiang Cui, Sakar Shivakoti, Z Hong Zhou

**Affiliations:** Department of Microbiology, Immunology and Molecular Genetics, University of California, Los Angeles (UCLA ), 609 Charles E Young Dr E, Los Angeles, CA 90095, USA; California NanoSystems Institute, UCLA, 570 Westwood Plaza Building 114 | Mail Code: 722710, Los Angeles, CA 90095, USA; Department of Chemistry and Biochemistry, UCLA, 607 Charles E. Young Drive East | Box 951569, Los Angeles, CA 90095-1569, USA; California NanoSystems Institute, UCLA, 570 Westwood Plaza Building 114 | Mail Code: 722710, Los Angeles, CA 90095, USA; Department of Microbiology, Immunology and Molecular Genetics, University of California, Los Angeles (UCLA ), 609 Charles E Young Dr E, Los Angeles, CA 90095, USA; Department of Microbiology, Immunology and Molecular Genetics, University of California, Los Angeles (UCLA ), 609 Charles E Young Dr E, Los Angeles, CA 90095, USA; California NanoSystems Institute, UCLA, 570 Westwood Plaza Building 114 | Mail Code: 722710, Los Angeles, CA 90095, USA

Dear Editor,

Aquareovirus (ARV, *Reoviridae*) causes hemorrhagic disease in the economically important golden shiner and grass carp of America and China, respectively (Nason et al., 2000; Fang et al., 2005). *Reoviridae* members are characterized by endogenous transcription of their multipartite genomes within capsids of 1–3 layers and are further classified based on the presence (*Spinareovirinae* subfamily, 9 genera) or absence (*Sedoreovirinae* subfamily, 6 genera) of mRNA-capping turrets along the innermost layer (King et al., 2012). The innermost layer of reoviruses is always an icosahedral, *T* = 2*, inner capsid particle (ICP) or core, which is transcriptionally competent (Farsetta et al., 2000). Among turreted reoviruses, cytoplasmic polyhedrosis virus (CPV) has a single-layered capsid, which is equivalent to the ICP within double- or triple-layered reoviruses (Hill et al., 1999; Zhang et al., 1999; Yu et al., 2011). This simple structural organization makes CPV an attractive model to study turreted reoviruses, but renders it inadequate to describe possible impacts of shedding the external layers from the numerous, multi-layered *Spinareovirinae* members (Zhang et al., 2022). Here we used a sequential symmetry expansion and relaxation approach to resolve the first asymmetric reconstruction of the ARV ICP by cryoEM to 3.3 Å (Fig. S2). Comparison with existing ARV virion and infectious subvirion particle (ISVP) structures (Ding et al., 2018) reveals expansion of the ICP and concomitant conformational changes to the transcription related proteins.

Lacking the outer capsid proteins VP5 and VP7, the ARV ICP retains the icosahedral, *T* = 2*, inner capsid shell composed of 60 asymmetric dimers of the 1214-residue, wedge-shaped, VP3 capsid shell proteins (CSPs) and 120 symmetrically arranged copies of the clamp protein (VP6) which form the ICP frame and provide support, respectively (Fig. 1A). VP3 dimers (containing conformers VP3_A_ and VP3_B_) encircle each 5-fold vertex; with VP3_A_ conformers seated around the 5-fold vertex center, creating pores adjacent to each transcriptional enzymatic complex’s (TEC’s) template exit channel for direct transcript capping and release via VP1 turret proteins (TPs) (Fig. 1C and 1D). VP3_B_ conformers partially intercalate between VP3_A_ monomers, and form 3-fold vertices with neighboring decameric assemblies (Fig. 1D). Absent conspicuous VP3 rearrangement, the internal volume of the ICP increased from 5.51 × 10^7^ Å^3^ to 6.02 × 10^7^ Å^3^, or about 9.3% relative to the grass carp reovirus (GCRV) virion and ISVP with which golden shiner reovirus (GSRV) shares 96%–100% a.a. sequence identity (Fig. 1B) (McEntire et al., 2003; Ding et al., 2018; Wang et al., 2018). By reducing the packaging density and thus viscosity of the genome, the enlarged ICP provides the rigid dsRNA segments greater freedom of movement and presumably reduces the energy required to initiate transcription (Demidenko and Nibert, 2009). The changes undergone by individual decamers includes a 10 Å rise away from the virion origin and subtle expansion of the CSPs (Movie S2). The observed expansion can be attributed to a non-uniform elongation of the CSP monomers approximately 6 Å radially from the icosahedral 5-fold (I5) vertices, relative to their coated counterparts and differs between VP3 conformers (Movies S1 and S3).

**Figure 1.**
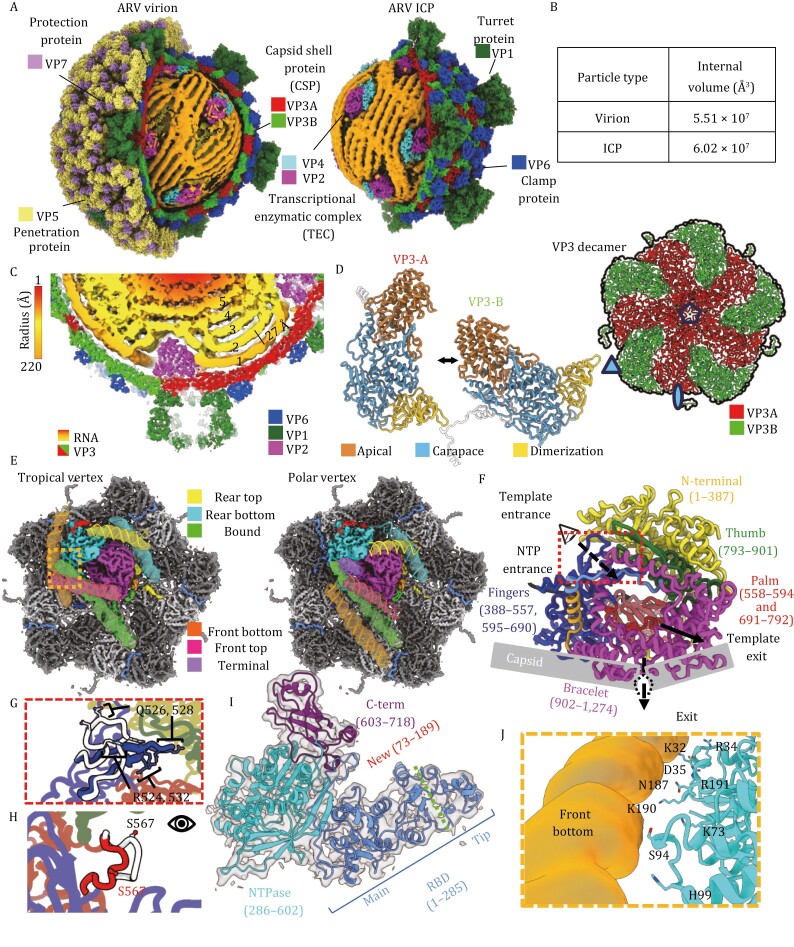
**Asymmetric reconstruction of ARV ICP reveals architectural changes to capsid shell and polymerase enzyme.** (A) CryoEM structures of ARV virion (left) (5VST) and ICP (right) with portion of their external layers removed along hemispheres to expose genome and TECs. (B) Table comparing volumetric difference between virion and ICP lumen. (C) Cross sectional view of Tropical Vertex featuring TEC and a turret. Layers of genome segments are numbered from 1–5 based on proximity to capsid wall. (D) Ribbon Diagram of ICP capsid decamer (top right) and VP3A (left) and VP3B (right) conformers with “tip” (a.a. 486–830), “carapace” (a.a. 190–485, 831–976, and 1,144–1,214), and “dimerization” (a.a. 977–1,143) domains indicated and colored differently. (E) Comparison of the CryoEM density maps from polar and tropical vertices, highlighting the TEC (magenta and cyan) and surrounding RNA, shown semi-transparently and superposed with corresponding dsRNA models (colored by segment) and labeled “Rear Top”, “Rear bottom”, “Bound”, “Front bottom”, “Front top”, and “terminal” based on position. (F–H) Atomic model of ICP RdRp VP2 colored by domain including the N-terminal domain (1–387), C-terminal bracelet (902–1,274), and core (388–901) colored by subdomain, including the thumb (793–901), fingers (388–557 and 595–690), and palm (558–594 and 691–792) (F). (G) and (H) views from (F) are shown to demonstrate the difference between the ICP and ISVP of the template channel finger loop (G) and catalytically important priming loop (H). (I) Atomic model of RdRp cofactor VP4 colored by domains with newly resolved residues indicated (red) and division of RBD into Main and Tip subdomains (green separator). (J) Interactions of the polar and charged residues from the newly resolved NTPase residues with the adjacent RNA segment from boxed region in E (orange).

Each ARV CSP is divided into three distinct domains which include the apical “tip” nearest the I5 vertices, the large carapace domain, and the small β-sheet rich dimerization domains (Fig. 1D). The local shifts of CSP dimers reveal a non-uniform elongation of the apical and carapace domains, with the alpha helices migrating away from the I5 vertices and towards the icosahedral two-fold and three-fold (I2 and I3) vertices for VP3_A_ and VP3_B_, respectively (Movie S2). In the apical domain helices 13 and 14 and their conjoining loop (a.a. 490–518), which line the I5 transcript exit channel at the luminal side of the capsid, are largely unperturbed by the capsid shifts and help to maintain a consistent pore diameter. By contrast, the other I5 adjacent elements of the apical domains move away from the I5 channel, and the helices 13 and 14 of VP3_**B**_ migrate in a similar manner as the other secondary structural elements. Closer inspection reveals striking conservation of VP3_A_ residues adjacent to the I5 pore (a.a. 490–518, RMSD 464 Å) when compared to the quiescent virion and ISVP structures (Ding et al., 2018; Wang et al., 2018). Despite exhibiting outward movement, the secondary structural elements of CSPs which interface with the clamp and turret proteins interfaces appear to move as rigid bodies, presumably constrained by interactions with the essential clamp and turret proteins (Movie S2).

Interactions of ARV inner and outer capsid proteins (VP3–VP5) are mediated through the clamp protein VP6, making it important for both stabilization against the genome and outer shell association. Previous work has shown the related mammalian orthoreovirus (MRV) ICPs can be recoated to form ISVPs (Farsetta et al., 2000), suggesting the ARV clamps remain in a VP5 receptive state following uncoating. Superimposition of ICP and virion clamp proteins reveal significant conformational similarity (RMSD 0.667 Å) despite their migration away from the I5 vertices in ICPs. While inconsequential for parental ARV particles, which irreversibly cleave VP5 during entry, this may provide a platform onto which VP5 can bind, compress nascent core particles, and halt transcription inside ARV progeny.

Within ICPs, several dsRNA segments interact with each TEC and these interactions stabilize segments, enabling improved visualization of their major and minor grooves as observed in other RNA viruses (Fig. 1E) (Pan et al., 2021). Five major dsRNA segments are observed adjacent to each TEC and are labeled based on their positions relative to the TEC, with a 6th segment observed adjacent to the template entrance in polar vertices (Fig. 1E). The VP2 RNA-dependent RNA polymerase (RdRp) is organized into the N-terminal domain (NTD), C-terminal bracelet (CTB), and RdRp core which is further differentiated into the thumb, fingers, and palm subdomains (Figs. 1F and S4) (Ding et al., 2018; Wang et al., 2018). The fingers house the NTP entry channel and, with the thumb, facilitate elongation and proofreading while the palm catalyzes phosphodiester bond formation between new NTPs and growing strands via the highly conserved D591, D740, and D741 residues (Fig. S4B). The polymerase possesses several channels to funnel RNA templates and transcription products while the CTB of the inactive ICP does not occlude the template exit channel as in quiescent CPV (Fig. S4D and Movie S3) (Ding et al., 2018).

Our RdRp structure reveals several local architectural changes within these RdRp channels when compared to the ISVP (Fig. 1F–H, and Movie S3). Viewed down the template entry channel, a positively charged finger domain loop extends into the template entry channel in the ICP, widening to accommodate nucleic acids (Fig. 1G, Movie S3). From within the channel, the priming loop—thought to separate template and transcript strands—shifts away from the transcript exit channel and orients the catalytically important serine residues towards the would-be incoming template (Fig. 1H, Movie S3). This migration away from the CTB widens the mRNA exit channel, likely promoting exit through the adjacent I5 pores and turrets. From the external TEC view, the RdRp expansion along the capsid lumen appears linked to the radial expansion of the capsid beneath it (Movie S3). This contrasts with the VP4 NTPase which undergoes a unidirectional shift, consistent with the radial expansion of its associated CSP monomers (Movie S3). These movements may be linked to the asymmetric association of the TEC along the expanding decameric subunit. As VP4 is situated primarily atop the VP3_1_ dimer it moves along with VP3_1_ dimer elongation, whereas the VP2, seated atop VP3_1–4_, is drawn in several directions based on uniform expansion of the capsid and the proportion of RdRp associated with each conformer pair (Fig. 2A and 2B). As uncoating is necessary to synthesize complete viral transcripts (Farsetta et al., 2000), these subtle conformational changes in the TECs described here may be essential to carrying out efficient viral transcription.

**Figure 2.**
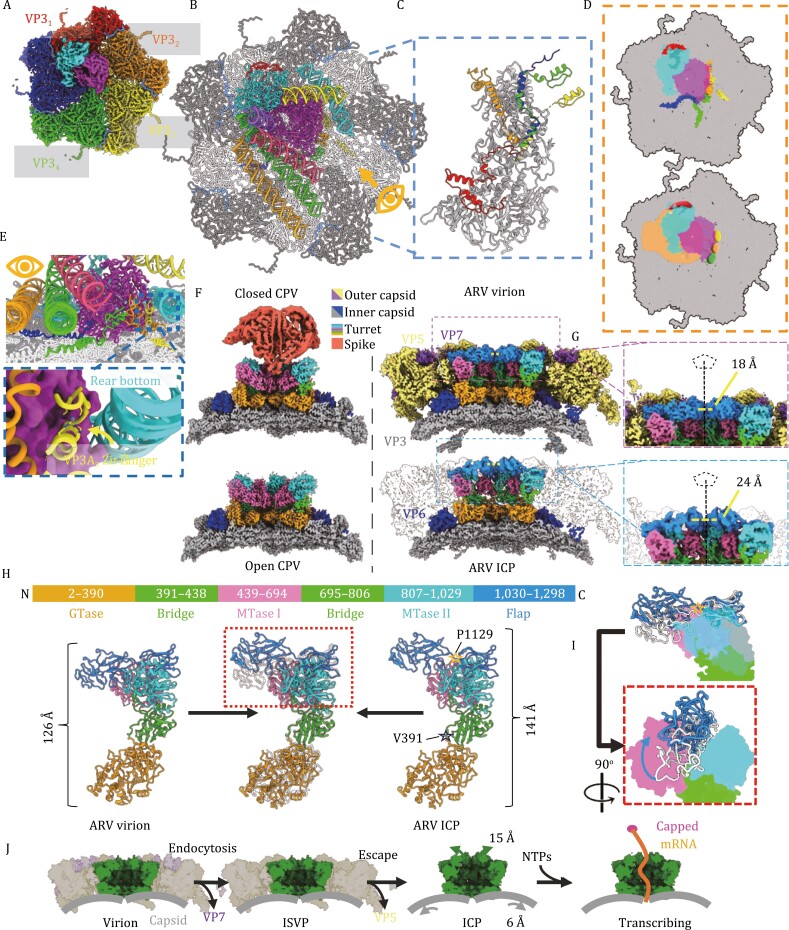
**Asymmetric interactions of VP3**_**A**_ **N-termini with TEC and nucleic acids and conformational changes of the capping enzyme turret in uncoated virus.** (A and B) CryoEM map of polar decamer and attached TEC (left) with dimer pairs colored based on TEC association (A), and corresponding atomic model (B). Note the RNA is only shown in the atomic model (B) and not shown in the cryoEM density for clarity. (C) Superposition of VP3_A_ proteins from each dimer pair, with N-terminal residues colored to match dimer pairs from (A). (D) Depiction of TEC along decamer lumen with N-terminal surfaces of VP3_A_ CSPs colored according to dimer pair in (A, left), and hand shape added to improve visualization of Zn-fingers grasping TEC. (E) Side views of atomic model as indicated by eye symbols in (B), with VP3_A_ N-termini colored as in (C), and magnified view of VP3_A3_ Zn-finger domain interacting with both RdRp (magenta) and rear bottom dsRNA (cyan). (F) comparison of CPV before and after detachment, where the Receptor binding spike remains attached to the turret and corresponding region of ARV virion and ICP. (G) Magnified view of turret pores from ARV virion (top) and ICP (bottom) with I5 symmetrical axis indicated with pentagon and line segment. (H) Atomic structure of the ARV turret protein VP1 from virion (left) and ICP (right) with superimposition of MTase domains (center) to show difference, with domains depicted (top) with the primary sequence number and corresponding color scheme. (I) magnified view of boxed region from (H), with solid color of non-flap domains used to highlight virion to ICP differences. (J) Schematic demonstrating the confirmational changes differences undergone by ARV turrets (green) from virion to ISVP and ICP, which coincide with the loss of outer capsid proteins VP5 and VP7 (translucent yellow and purple).

Situated beneath 11 of 12 I5 vertices in ARV are TECs, heterodimers of RdRp and NTPase (Figs. 1A and S3A). NTPase has an RNA-binding domain (RBD) with its “tip” and “main” subdomains, an NTPase domain, and a C-terminal domain (CTD) (Figs. 1I and S5) (Ding et al., 2018). The previously missing *tip* and much of the *main* subdomains are now observed extending away from the TEC core towards the template exit channel (Fig. S5). This separation may accommodate dynamic RNA interactions throughout transcription. The newly modeled N-terminal residues also reveal a flexible region homologous to that of ARV’s MRV cousin but of a distinct fold (Pan et al., 2021), with extensive genome segment interactions (Fig. 1J).

The N-terminal residues of VP3_A_ conformers were shown to associate with and lie along the exterior of the TEC, on both RdRp and NTPase (Ding et al., 2018), and were suggested to anchor the TEC into place. The newly modeled VP3_A_ N-terminal residues include the previously unresolved residues 108–152 (VP3_A1–4_) which contain Zn-finger domains (a.a. 116–141) (Fig. 2B and 2E), and conform to a traditional Cys_2_His_2_ nucleic acid binding motif (Fig. 2E) (Yu et al., 2011) Here four newly resolved Zn-fingers (VP3_A1–4_) lie along the TEC, with VP3_A2–4_ situated along an RdRp cleft opposite the NTPase, and VP3_A1_ seated along the VP4 NTPase domain forming a four-pronged setting that anchors the TEC complex to the capsid shell (Fig. 2D). The VP3_A3_ Zn-finger also contacts the rear bottom genome segment (Fig. 2E), which suggests involvement in transcription initiation as observed in rotavirus (Ding et al., 2019). ARV VP3s are thus multifunctional, promoting TEC assembly and stability while maintaining genome organization in the quiescent particles. Despite expansion of the capsid shell, the Zn-fingers are positioned as in ISVP, suggesting the Zn-fingers function independent of the capsid shell. This may be enabled by the flexible linker within the CSP N-terminal domains (a.a. 142–190), which extend to accommodate capsid expansion, while maintaining their TEC association and without altering genome organization or TEC activation state (Movie S3).

Atop each I5 vertex sits a pentameric turret, composed of five copies of VP1 TPs (Figs. 1A and 2F) with their axial channels reaching the nascent mRNA translocating pores of the TEC (Reinisch et al., 2000). When ARV ICP and virion turrets are compared, we observe an axial extension of the TPs and widening of the axial channels (Fig. 2G and 2H). Local alignment of the 5 TP domains reveals the enzymatic guanylyltransferase (GTase) and methyltransferase I & II (MTase I & II) domains remain stable (RMSD 0.842 Å and 0.907 Å respectively) while domain separation occurs along the flexible linker regions between the GTase, MTase, and flap domains (Fig. 2H). This separation is enabled by the absence of the VP5 layer that would otherwise clash with the ICP turret conformation (Fig. 2G). Despite MRV possessing a similar double layered architecture, analogous TP shifts are not observed when the MRV ICP and complete virions are compared (Reinisch et al., 2000; Pan et al., 2021).

CPV and MRV virions both have spike proteins which occupy the turret channel and serve dual roles, mediating cell entry, and preventing premature transcript escape (Fig. 2F) (Pan et al., 2021; Zhang et al., 2022). As ARV lacks a spike protein homologue, we investigated the flap domains that line the turret exit channel. From virion to ICP, the two C-terminal IG domains undergo an outward shift of 14 Å and counterclockwise twist when viewed from within the I5 channel and originates at the flexible linker region (a.a. 1,129) (Fig. 2I). This finding, coupled with the changes to capsid and TEC architecture, suggests a regulatory mechanism of transcription wherein uncoating yields a particle whose capsid better accommodates genome transcription and whose capping enzymes are widened, or primed, for transcript export.

In summary, this work provides the first near-atomic resolution asymmetric reconstruction of an ICP from a turreted, multi-layered reovirus particle. The structure reveals subtle but functionally important conformational changes compared to the structures of coated ARV. These changes in the internal capsid volume, along-side a widening of TEC nucleotide channels and extension of the 5ʹ-cap synthesizing turret proteins create an architectural environment conducive to endogenous transcription. Therefore, the specialized outer capsid layers serve as a useful transcriptional regulator, ensuring transcription requires not only the presence of cofactors, but also loss of outer capsid layers which occurs upon cell entry.

## Supplementary information

The online version contains supplementary material available at https://doi.org/10.1093/procel/pwad002.

pwad002_suppl_Supplementary_Material

pwad002_suppl_Supplementary_Movies

## Footnotes

This work was supported in part by grants from the National Institutes of Health (AI094386 to Z.H.Z.). A.S. received support from NIH Ruth L. Kirschtein National Research Service Award AI007323. We acknowledge the use of resource at the Electron Imaging Center for Nanomachines supported by UCLA and by instrumentation grants from NIH (1S10RR23057, 1S10OD018111 and U24GM116792) and NSF (DBI-1338135 and DMR-1548924) and at the UCLA AIDS Institute supported in part by UCLA-CDU CFAR (NIH AI152501), the James B. Pendleton Charitable Trust and the McCarthy Family Foundation. Z. Hong Zhou, Alexander Stevens, Yanxiang Cui, and Sakar Shivakoti declare no conflict of interest. This article does not contain any studies with human or animal subjects performed by the any of the authors. Z. Hong Zhou, Alexander Stevens, Yanxiang Cui, and Sakar Shivakoti reviewed and approved the paper. CryoEM maps of the asymmetric GSRV ICP and sub-particles have been submitted to the Electron Microscopy Data Bank and can be found under accession numbers EMD-29337, EMD-29243, EMD-29244, and EMD-29243 for the cryoEM structures of the complete capsid, polar vertex, tropical vertices with and without TECs respectively. The coordinates of ICP polar and tropical vertex sub-particles were deposited in the Protein Data Bank under accession number 8FJK and 8FJL. Z.H.Z. designed and supervised the project; S.S. prepared sample and made cryoEM grids; Y.C. performed cryoEM imaging and 3D reconstruction; A.S. built the atomic models, interpreted the structures, made the figures, and wrote the paper with input from Z.H.Z.; All authors reviewed and approved the paper.
