# Supplementary material for: Asymmetric reconstruction of the aquareovirus core at near-atomic resolution and mechanism of transcription initiation
Source: Protein Cell. 2023 Feb 4;14(7):546–50. doi: 10.1093/procel/pwad002 (PMC10305738; doi:10.1093/procel/pwad002)
Supplement: pwad002_suppl_Supplementary_Material [file pwad002_suppl_supplementary_material.pdf]

Asymmetric reconstruction of the aquareovirus core at near-atomic resolution and mechanism if transcription initiation

Alexander Stevens, Yanxiang Cui, Sakar Shivakoti, Z. Hong Zhou*

Supplementary Information

Methods and Data Availability

Viral Culture and Isolation

Golden shiner reovirus stock was purchased from ATCC (ATCC® VR957™) and was propagated in Fat Head Minnow cells (FHM; ATCC® CCL-42™) with a slight modification to previously described method (Shaw et al., 1996). FHM cells were cultured in MEM, hanks' balanced salts (Gibco™) supplemented with 10% fetal bovine serum (Atlanta biologicals), Penicillin (10,000 IU) and Streptomycin (10,000 µg/ml) (Corning ®, USA), and maintained at 28°C. Confluent monolayers of FHM were infected with ~5 plaque forming units of GSRV per cell for ~48 hours. Once cell syncytia was visible, media was collected from the infected cell culture and centrifuged at 11,000 g for 15 min to get rid of cells and cellular debris. Clean supernatant was centrifuged at 100,000g for 3 hours (SW28 rotors, Beckman Coulter) to pellet the viral particles. Supernatant was discarded and 100 µl of ice-chilled PBS buffer was added to each pellet to resuspend the viral particles overnight. Pellets were pooled and loaded on a 15%-50% sucrose gradient and banded by centrifuging at 100,000 g for 1 hour (SW41 rotor) at 4°C. Band containing the GSRV cores was harvested and diluted in PBS, pelleted at 100,000 g for 2 hours. The cores in the pellet were resuspended in PBS and quality confirmed by negative-staining (2% uranyl acetate) stain transmission electron microscopy.

CryoEM imaging

For cryoEM sample preparation, 2.5-µL aliquots of the GSRV core sample were applied to 200-mesh Quantifoil grid with 2µm-diameter hole size (2/2 or 2/1 Quantifoil) and plunge frozen in a Vitrobot Mark IV (Thermo Fisher Scientific) (Thermo Fisher Scientific) after blotting (blot time 15 seconds, blot force -5, 100% humidity and 4°C). Movies of dose fractionated image frames were recorded using a Titan Krios microscope (Thermo Fisher Scientific) equipped with a Gatan imaging filter (GIF) quantum LS and a post-GIF Gatan K2 Summit direct electron detector

operated in super resolution mode with a calibrated pixel size of 0.68 Å/pixel with SerialEM (Mastronarde, 2005). The GIF slit width was set to 20 eV at the zero-loss peak, and dose rate as recorded by the camera was set to with a total exposure dosage of ~45 e/Å^2 at the sample level which was fractionated equally into 40 frames. A defocus range of 1.5μm-2.5 μm was targeted for recordings. Dose-fractionated frames were binned 2× (effective pixel size 1.36 Å) and aligned for beam induced motion correction to produce dose-weighted and unweighted micrographs, used for final reconstruction and initial screening and CTF estimation respectively, using MotionCor2 (Zheng et al., 2017). In total 12,258 movies were recorded.

Data processing

Defocus values of the micrographs were initially estimated using CTFFIN4 (Rohou and Grigorieff, 2015), and those micrographs with significant ice contamination or defocus values beyond -0.8 and -3.0 μm were removed leaving 10,935 micrographs. 38,486 particles were initially selected by Ethan (Kivioja et al., 2000). The particles were extracted and initially subjected to an icosahedral refinement in RELION which yielded a 3.4 Å cryoEM map of the complete viral particle (Scheres, 2012). In our icosahedral reconstruction, strong densities were observable beneath the positions of I5 vertices. We then followed a previously established stepwise symmetry relaxation workflow within RELION to generate asymmetric sub-particle reconstructions of both polar and tropical vertices (Ding et al., 2018; Sjors H.W. Scheres, 2012; Zhang et al., 2015). Briefly, particles from the 3.4 Å icosahedral reconstruction were subjected to symmetry expansion (option I3) using the *relion_symmetry_expand* command, to generate a RELION STAR file containing 60 orientation entries for each ARV particle, corresponding to 5 copies of each I5 vertex differentiated by the rotation about the I5 vertex, and listed as _rlnAngleRot. One entry corresponding to each vertex was kept, yielding a duplicate-entries-removed STAR file with ~12 entries, or one for each vertex, for each ICP. The icosahedral reconstruction orients in such a way that the location of one vertex consistently lies along the *z* axis, so we performed classification on ICP particles with the

capsid density subtracted to identify tropical and polar vertices and estimated their location for extraction of the penton sub-particles using the RELION “Particle extraction” tool (Sjors H W Scheres, 2013). Polar and Tropical vertices were separately refined into the final sub-particle maps provided here. Unfortunately, this method failed to resolve the true asymmetric structure featuring 11 TECs, as the pseudo-D3 symmetrical particle featured 12 TECs, hindering efforts to resolve the unoccupied vertex. This was overcome following the D3-symmetry expansion of the ICP particles, generating 6 duplicates within a new STAR file. The particles were then subjected to exhaustive classification with a reference mask beneath a tropical vertex to identify a class without a TEC. Using the orientations determined during this classification we were able to reconstruct the complete asymmetric ICP.

Atomic Model Building and Refinement

Atomic models of the GSRV RdRp, CSP, NTPase, turret and clamp proteins were initially modeled into the cryoEM density in Coot using the GCRV ISVP (PDB 6M99) as a homology following previously established protocols (Emsley et al., 2010; Yu et al., 2018; Ding et al., 2018). Following initial fitting, the polypeptide chains were mutated to match the GSRV sequence using Coot’s Mutate Residue Range tool prior to local and global refinement in PHENIX (Afonine et al., 2018). Residues were built into previously unresolved residues, de novo using Coot, except for the first 200 residues of VP4, whose structure was predicted in AlphaFold2 prior to fitting into the cryoEM map (Jumper et al., 2021). Upon initial fitting the model was refined within the sub-particle map using the molecular dynamic flexible fitting (MDFF) feature in ISOLDE from the UCSF ChimeraX user interface (Goddard et al., 2018; Croll, 2018). Models were then subjected to a final round of PHENIX real space refine and the resulting structure was validated by the worldwide protein data bank validation server (Afonine et al., 2018; Berman et al., 2003). Root mean squared deviations (RMSDs) between protein chains were calculated using the matchmaker tool in UCSF ChimeraX. Internal capsid volume was calculated using the ChimeraX recipe “Measure volume

enclosed by a virus capsid" on our ARV ICP and the previously published virion structure (EMD-6969) (Wang et al., 2018). TEC distribution maps were generated using UCSF Chimera (Pettersen et al., 2004).

Modeling of dsRNA.

We inserted poly-AU, A-form dsRNA segments from a previously published MRV model (PDB 7ELH) into our cryoEM maps (Pan et al., 2021). Segments were roughly placed into the cryoEM map, and segments were further fit in ISOLDE (Croll, 2018). Briefly, adaptive distance restraints were applied to the nucleic acid segments with reduced weight applied to the non-crystallographic map were fit into the cryoEM maps using ISOLDEs MDFF feature (Croll, 2018).

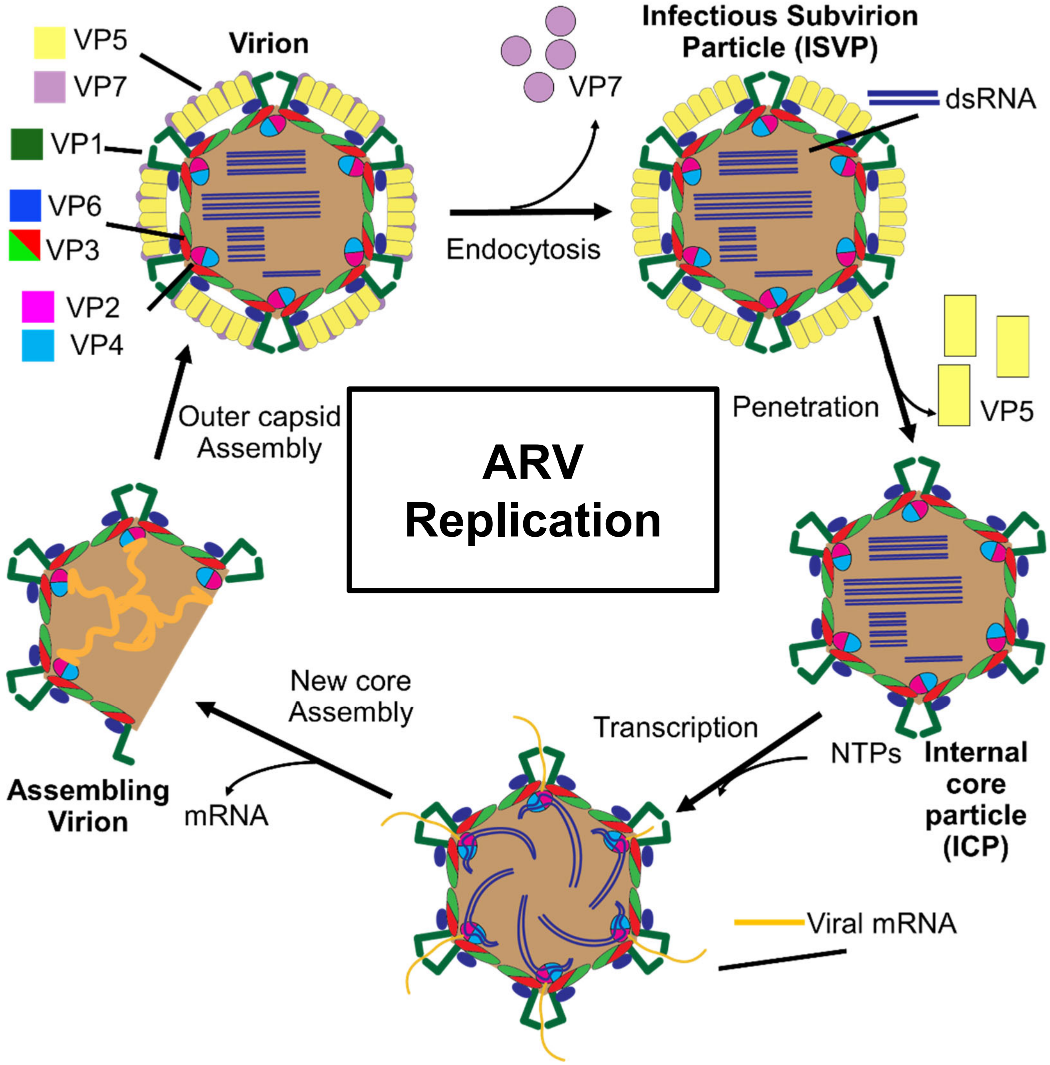

Figure S1. Typical changes to Aquareovirus capsid during replication. Diagrammatic representation of an Aquareovirus in cross section and the proposed replication process. The identities of viral structural and enzymatic proteins are indicated using the nomenclature for *Aquareoviruses* and assigned colors. Double-stranded DNA is represented by parallel blue line segments and single-stranded RNA is represented by orange lines.

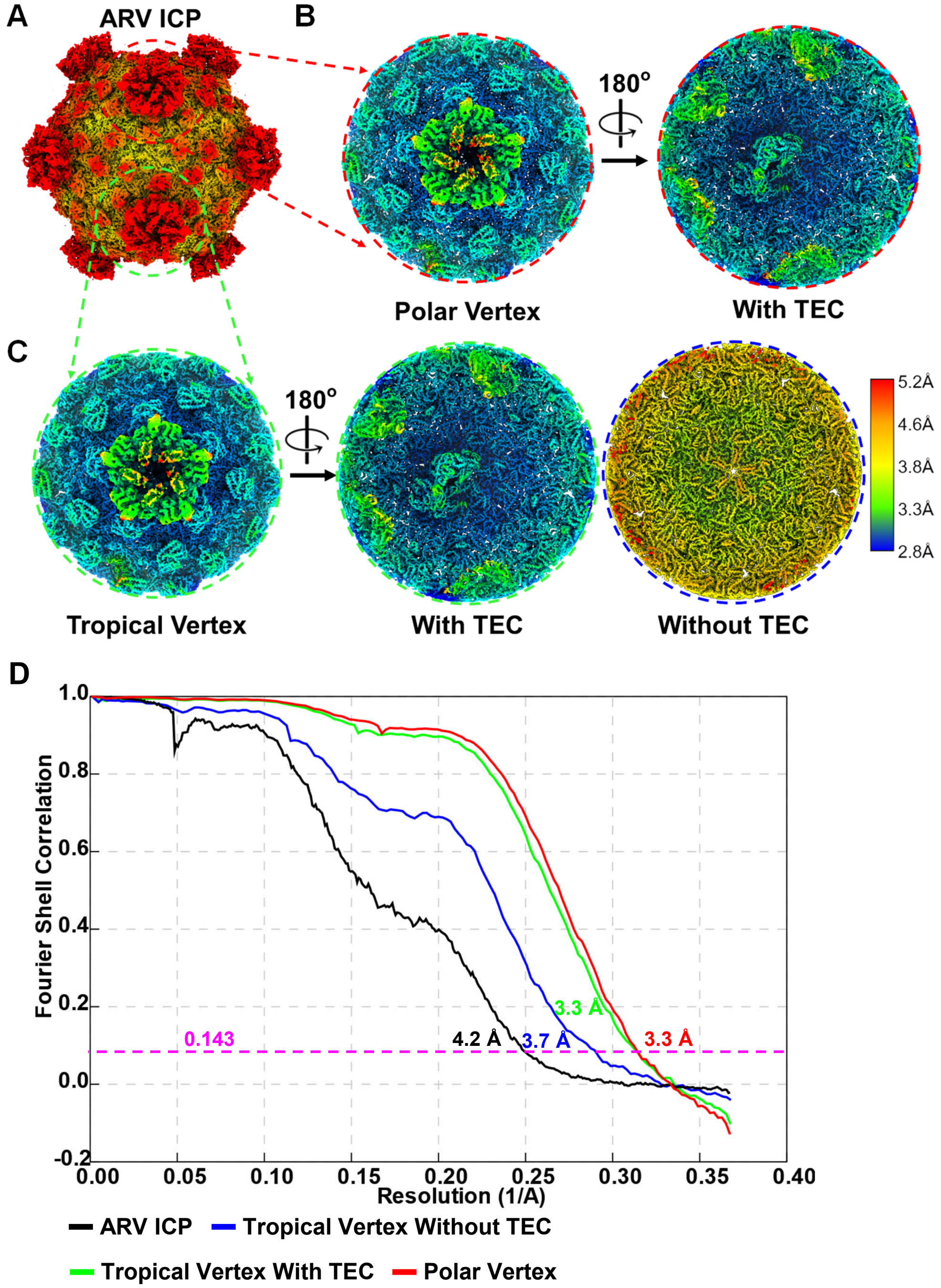

Figure S2. Asymmetric reconstructions from ARV ICP. (A-C) CryoEM maps of GSRV ICP (A) and sub particles from Polar (B) and Tropical (C) vertices colored by local resolution. Internal views of Polar and Tropical vertices in B and C (right and middle panels respectively) showing the asymmetrically associated TEC in both species and the lack thereof in a subset of Tropical vertices (C, right). (D) Gold-standard Fourier shell correlation (FSC) curves calculated from the independently refined half-maps of each cryoEM reconstruction in A-C, with resolution values listed for each species at FSC = 0.143.

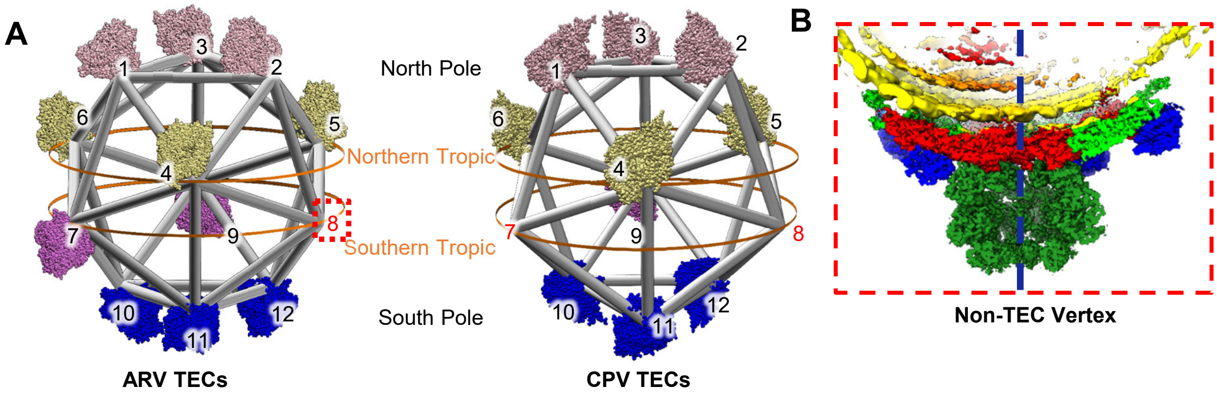

Figure S3. Asymmetric distribution of TECS within ARV ICP. (A) Position and orientation of the 11 ICP TECs relative to an icosahedron (grey) with Northern and Southern “poles” and “tropics” indicated. For clarity, RdRp distribution model not drawn to scale. (B) Cross sectional view of ICP vertex without TEC showing that the TEC-free vertex is occupied by RNA density (yellow).

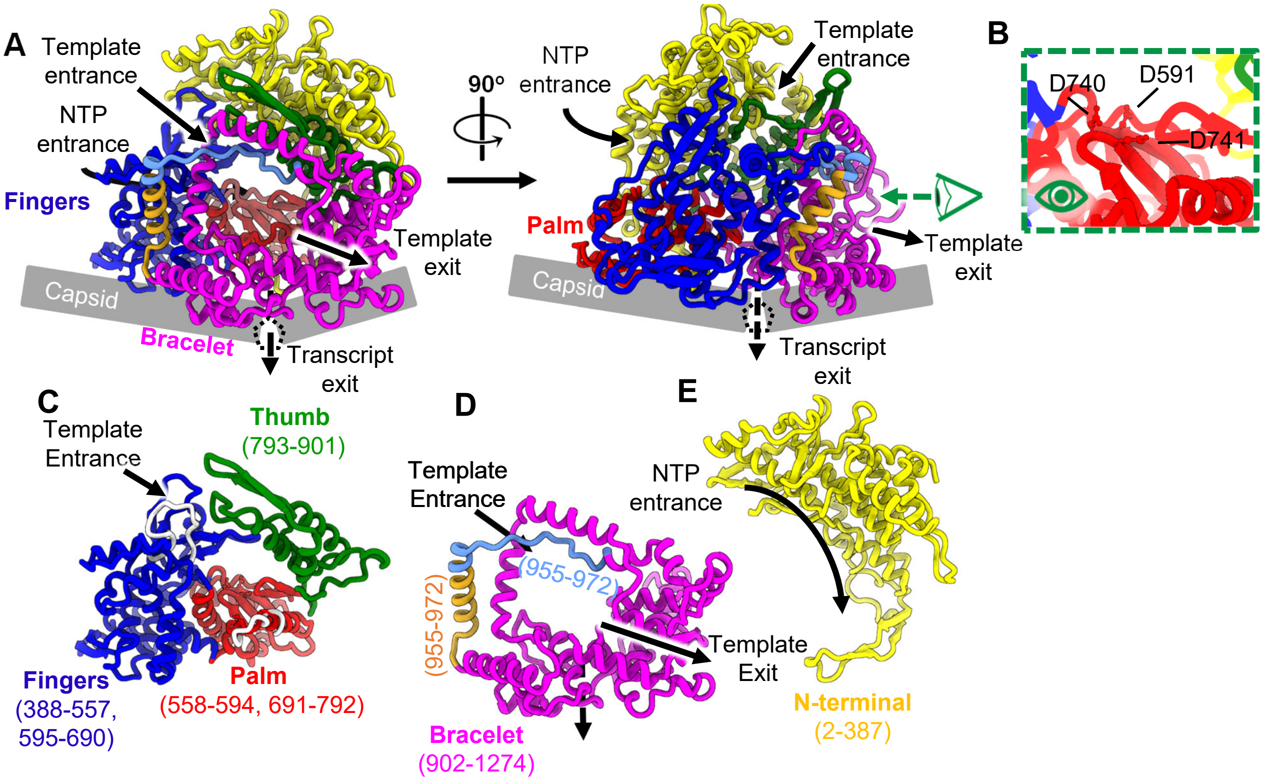

Figure S4. Exploded diagram of ARV ICP polymerase VP2. (A-E) Atomic model of ICP RdRp VP2 colored by domain, either shown together (A), or as an exploded view (C-E) to detail features. (C) provides magnified view of catalytic palm domain from A, with catalytically important residues indicated.

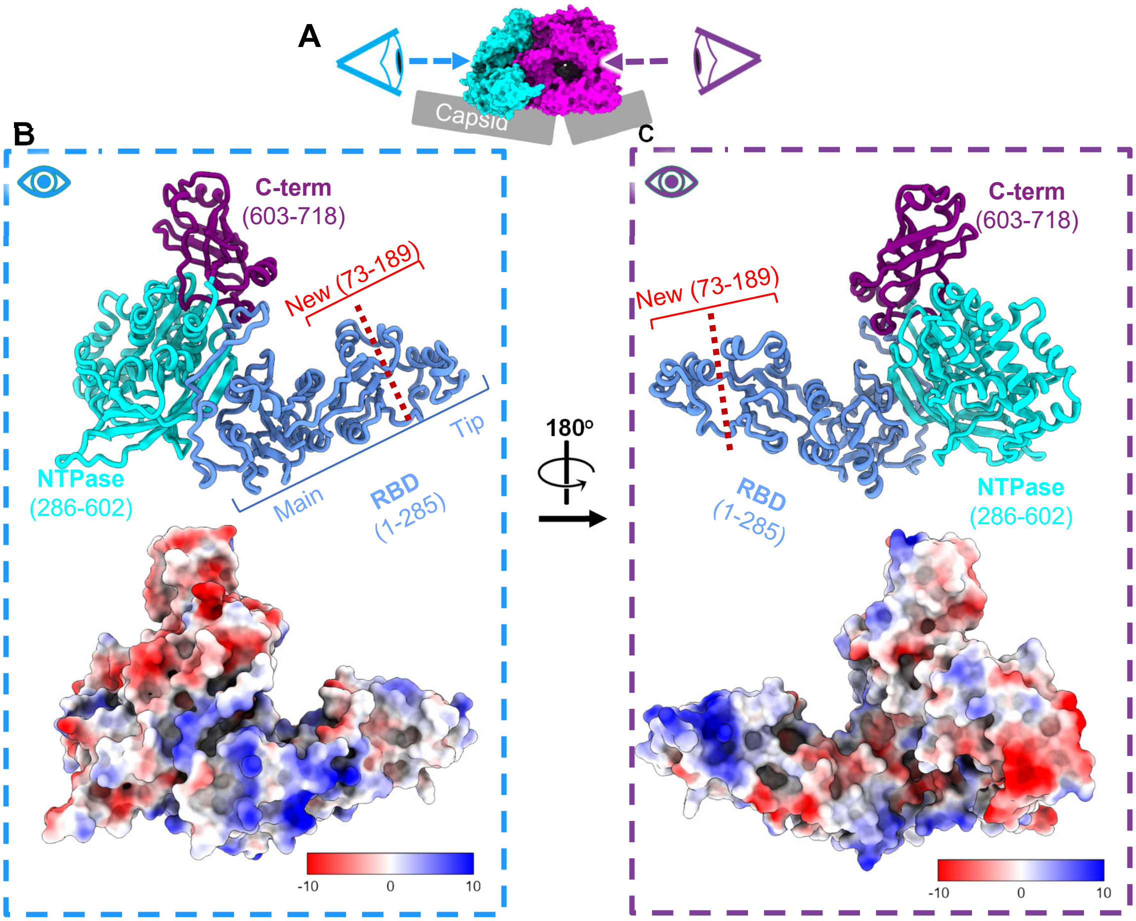

Figure S5. Columbic potential maps of NTPase VP4. (A-C) different views of NTPase VP4 protein depicted by ribbon diagrams and colored by domains (B-C, top). (B) View of NTPase as viewed along the face opposite the associated RdRp with surface colored by columbic potential (bottom). (C) View of the RdRp associated face of NTPase depicted in the same manner as B.

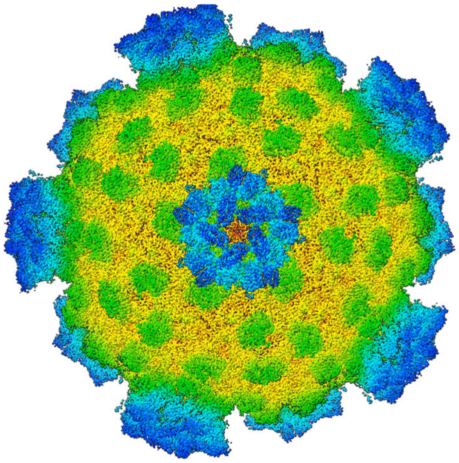

Movie S1. Expansion of ARV ICP decamer. Comparison of cryoEM maps and atomic models from core of ARV virion particle and ICP. Cartoon representation of ARV ICP, without TEC, conforming to same color scheme used in Fig. 1A. First morph movie demonstrates rise away from capsid center. Second morph toggles between virion and ICP decamers, superimposed at residues adjacent to the I5 vertices, from internal, side and external view to highlight conformational changes of capsid shell proteins (lime and red) and capping enzyme proteins (dark green). Rising motion away from virion origin not shown.

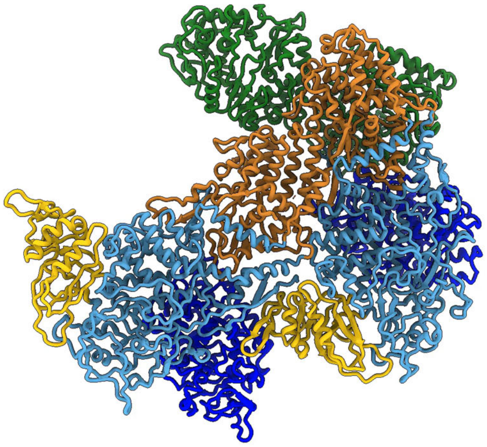

Movie S2. Local motion of CSP dimer from Virion to ICP. Cartoon representation of CSP dimer as colored in Fig. 1D, with local VP6 clamp proteins and the first 390 residues of the Turret protein VP1. Demonstrates local motion of VP3 monomers morphing between virion and ICP conformations. Side view demonstrates radial extension of $VP3_A$ and $VP3_B$ with the conserved position of $VP3_A$ α-helices 13 and 14. External view shows radial movement of clamp and turret proteins along external wall of ICP while their removal demonstrates the constrained movement of their interfaces during the morph.

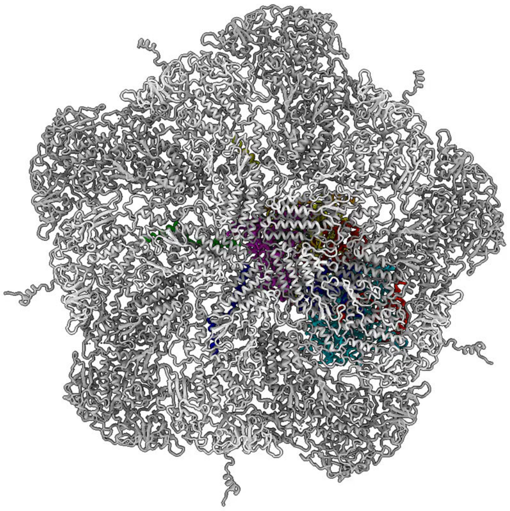

Movie S3. Conformational changes from ISVP to ICP TEC. Cartoon representation of decamer and TEC morphs between ISVP and ICP, demonstrating asymmetric movement of TEC, with NTPase moving along with capsid lumen while the RdRp appears to expand, creating a breathing like motion. Movie also emphasizes changes to RdRp finger loop at template entry channel and priming loop retraction from the transcript exit channel.

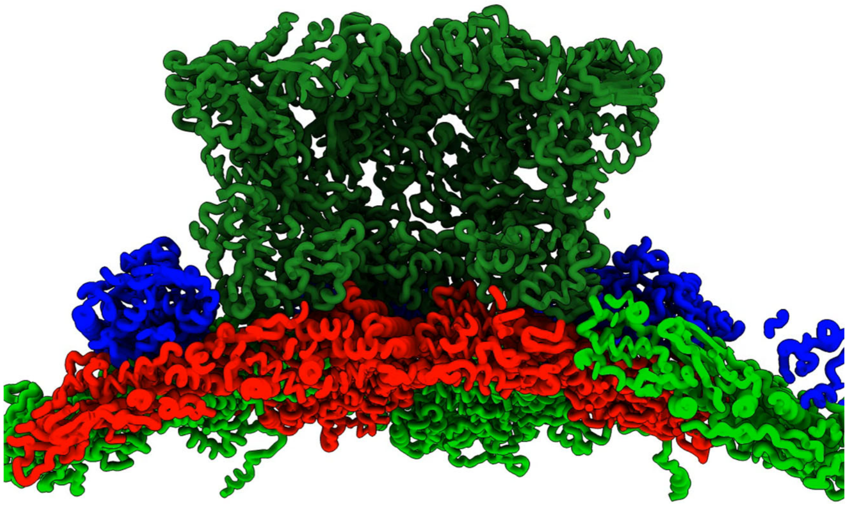

Movie S4. Extension of Turret proteins. Cross-sectional view of ARV turret proteins (dark green), CSPs (red and lime green), and clamp proteins (blue) morphing between ISVP and ICP confirmations and positions relative to the origin of the complete particle. Movie begins with ISVP decamer represented as cartoons morphing to ICP conformations.

Supplementary Table 1 Cryo-EM data collection, refinement, and validation statistics

	ICP Asymmetric reconstruction (EMD-29337)	Polar vertex with TEC (EMD-29243) (PDB 8FJK)	Tropical Vertex with TEC (EMD- 29244) (PDB 8FJL)	Tropical Vertex without TEC (EMD- 29336)
**Data collection and processing**				
Magnification	81,000	81,000	81,000	81,000
Voltage (kV)	300	300	300	300
Electron exposure (e–/Å^2)	45	45	45	45
Defocus range (μm)	-1.5 to -2.5	-1.5 to -2.5	-1.5 to -2.5	-1.5 to -2.5
Pixel size (Å)	1.36	1.36	1.36	1.36
Symmetry imposed	C1	C1	C1	C1
Initial particle images (no.)	36,764	36,764	36,764	36,764
Final particle images (no.)	15,433	119,182	99,323	21,844
Map resolution (Å)	4.2	3.3	3.3	3.7
FSC threshold	0.143	0.143	0.143	0.143
**Refinement**				
Initial model used (PDB code)		PDB 6M99	PDB 6M99	
Model resolution (Å)		3.0	2.9	
FSC threshold		0.143	0.143	
Model resolution range (Å)				
Map sharpening *B* factor (Å^2)		-100	-100	
<u>Model composition</u>				
Non-hydrogen atoms		183,951	183,948	
Protein residues		23,916	23916	
RNA Nucleotides				
Ligands		4	4	
<u>R.m.s. deviations</u>				
Bond lengths (Å)		0.006	0.006	
Bond angles (°)		1.000	1.000	
<u>Validation</u>				
MolProbity score		1.39	1.39	
Clashscore		3.67	3.67	
Poor rotamers (%)		0.53	0.53	
<u>Ramachandran plot</u>				
Favored (%)		96.47	96.47	
Allowed (%)		3.52	3.52	
Disallowed (%)		0.00	0.00	

References

Afonine, P. V., Poon, B. K., Read, R. J., Sobolev, O. V., Terwilliger, T. C., Urzhumtsev, A., et al. 74 (2018) 'Real-space refinement in PHENIX for cryo-EM and crystallography' *Acta Crystallographica Section D: Structural Biology*. International Union of Crystallography, pp. 531-544.

Berman, H., Henrick, K., & Nakamura, H. 10 (2003) 'Announcing the worldwide Protein Data Bank' *Nature Structural & Molecular Biology*. pp. 980-980.

Croll, T. I. 74 (2018) 'ISOLDE: A physically realistic environment for model building into low-resolution electron-density maps' *Acta Crystallographica Section D: Structural Biology*. International Union of Crystallography, pp. 519-530.

Ding, K., Nguyen, L., & Zhou, Z. H. 92 (2018) 'In Situ Structures of the Polymerase Complex and RNA Genome Show How Aquareovirus Transcription Machineries Respond to Uncoating' S. López *Journal of Virology*. pp. e00774-18.

Emsley, P., Lohkamp, B., Scott, W. G., & Cowtan, K. 66 (2010) 'Features and development of Coot' *Acta Crystallographica Section D Biological Crystallography*. International Union of Crystallography, pp. 486-501.

Goddard, T. D., Huang, C. C., Meng, E. C., Pettersen, E. F., Couch, G. S., Morris, J. H., et al. 27 (2018) 'UCSF ChimeraX: Meeting modern challenges in visualization and analysis' *Protein Science*. pp. 14-25.

Jumper, J., Evans, R., Pritzel, A., Green, T., Figurnov, M., Ronneberger, O., et al. 596 (2021) 'Highly accurate protein structure prediction with AlphaFold.' *Nature*. Springer US, pp. 583-589.

Kivioja, T., Ravantti, J., Verkhovsky, A., Ukkonen, E., & Bamford, D. 131 (2000) 'Local average intensity-based method for identifying spherical particles in electron micrographs.' *Journal of structural biology*. pp. 126-34.

Mastronarde, D. N. 152 (2005) 'Automated electron microscope tomography using robust prediction of specimen movements' *Journal of Structural Biology*. pp. 36-51.

Pan, M., Alvarez-Cabrera, A. L., Kang, J. S., Wang, L., Fan, C., & Zhou, Z. H. 12 (2021) 'Asymmetric reconstruction of mammalian reovirus reveals interactions among RNA, transcriptional factor μ2 and capsid proteins' *Nature Communications*. Springer US, pp. 1-16.

Pettersen, E. F., Goddard, T. D., Huang, C. C., Couch, G. S., Greenblatt, D. M., Meng, E. C., et al. 25 (2004) 'UCSF Chimera-A visualization system for exploratory research and analysis' *Journal of Computational Chemistry*. pp. 1605-1612.

Rohou, A., & Grigorieff, N. 192 (2015) 'CTFFIND4: Fast and accurate defocus estimation from electron micrographs' *Journal of Structural Biology*. Elsevier Inc., pp. 216-221.

Scheres, S. H. W. 180 (2012) 'RELION: Implementation of a Bayesian approach to cryo-EM structure determination' *Journal of Structural Biology*. Elsevier Inc., pp. 519-530.

Scheres, S. H. W. (2013) 'Single-particle processing in RELION' *Manuals*. pp. 1-21.

Shaw, A. L., Samal, S. K., Subramanian, K., & Prasad, B. V. 4 (1996) 'The structure of aquareovirus shows how the different geometries of the two layers of the capsid are reconciled to provide symmetrical interactions and stabilization' *Structure*. pp. 957-967.

Wang, X., Zhang, F., Su, R., Li, X., Chen, W., Chen, Q., et al. 115 (2018) 'Structure of RNA polymerase complex and genome within a dsRNA virus provides insights into the mechanisms of transcription and assembly' *Proceedings of the National Academy of Sciences of the United States of America*. pp. 7344-7349.

Yu, I., Nguyen, L., Avaylon, J., Wang, K., Lai, M., & Zhou, Z. H. 204 (2018) 'Building atomic models based on near atomic resolution cryoEM maps with existing tools' *Journal of Structural Biology*. Elsevier, pp. 313-318.

Zhang, X., Ding, K., Yu, X., Chang, W., Sun, J., Zhou, Z. H., et al. 527 (2015) 'In situ structures of the segmented genome and RNA polymerase complex inside a dsRNA virus' *Nature*. Nature Publishing Group, pp. 531-534.

Zheng, S. Q., Palovcak, E., Armache, J.-P., Verba, K. A., Cheng, Y., & Agard, D. A. 14 (2017) 'MotionCor2: anisotropic correction of beam-induced motion for improved cryo-electron microscopy' *Nature Methods*. pp. 331-332.
