# Supplementary material for: Asymmetric reconstruction of the aquareovirus core at near-atomic resolution and mechanism of transcription initiation
Source: Protein Cell. 2023 Feb 4;14(7):546–50. doi: 10.1093/procel/pwad002 (PMC10305738; doi:10.1093/procel/pwad002)
Supplement: pwad002_suppl_Supplementary_Movies [file pwad002_suppl_supplementary_movies.pptx]

## Slide 1
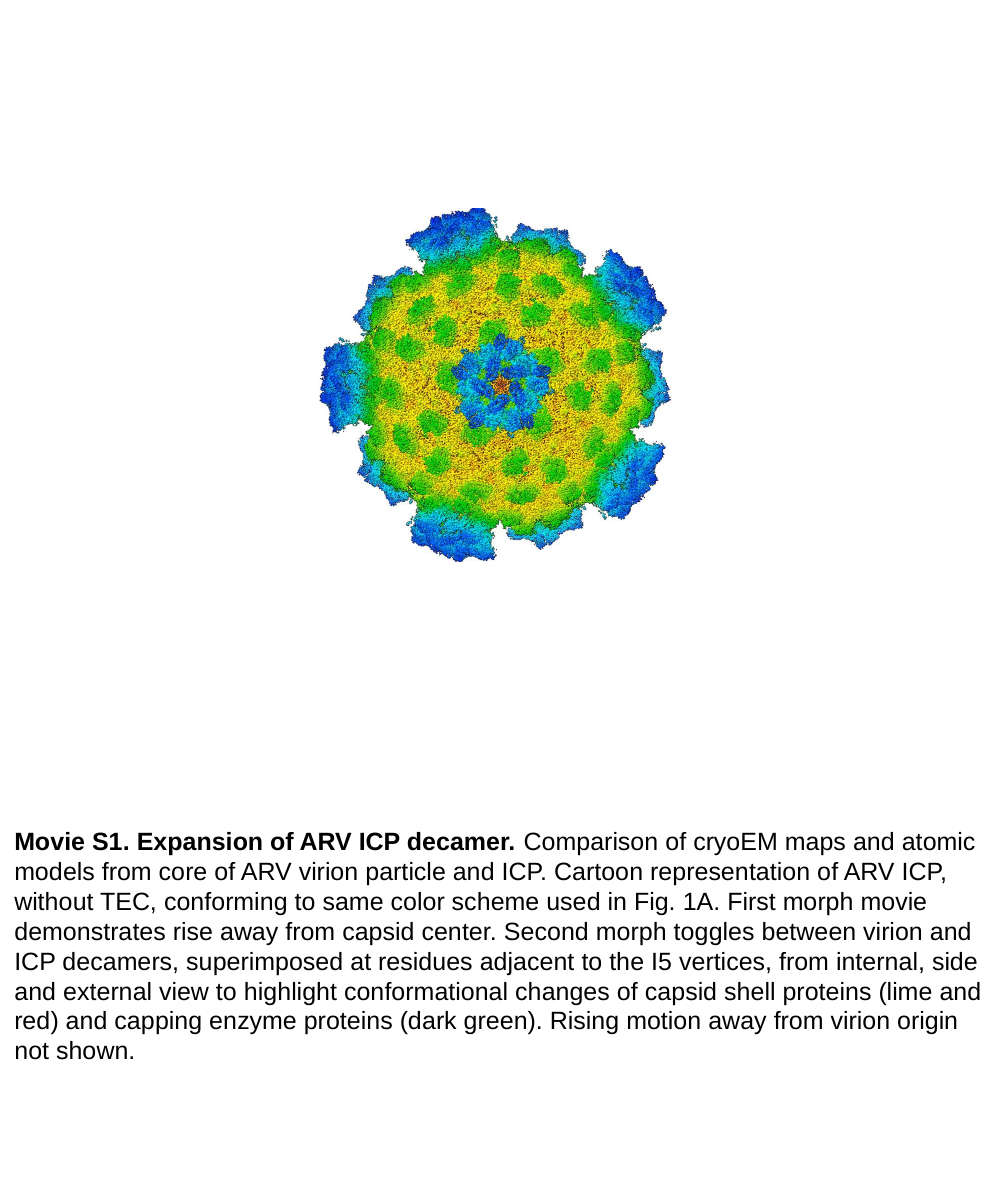

Movie S1. Expansion of ARV ICP decamer. Comparison of cryoEM maps and atomic models from core of ARV virion particle and ICP. Cartoon representation of ARV ICP, without TEC, conforming to same color scheme used in Fig. 1A. First morph movie demonstrates rise away from capsid center. Second morph toggles between virion and ICP decamers, superimposed at residues adjacent to the I5 vertices, from internal, side and external view to highlight conformational changes of capsid shell proteins (lime and red) and capping enzyme proteins (dark green). Rising motion away from virion origin not shown.

## Slide 2
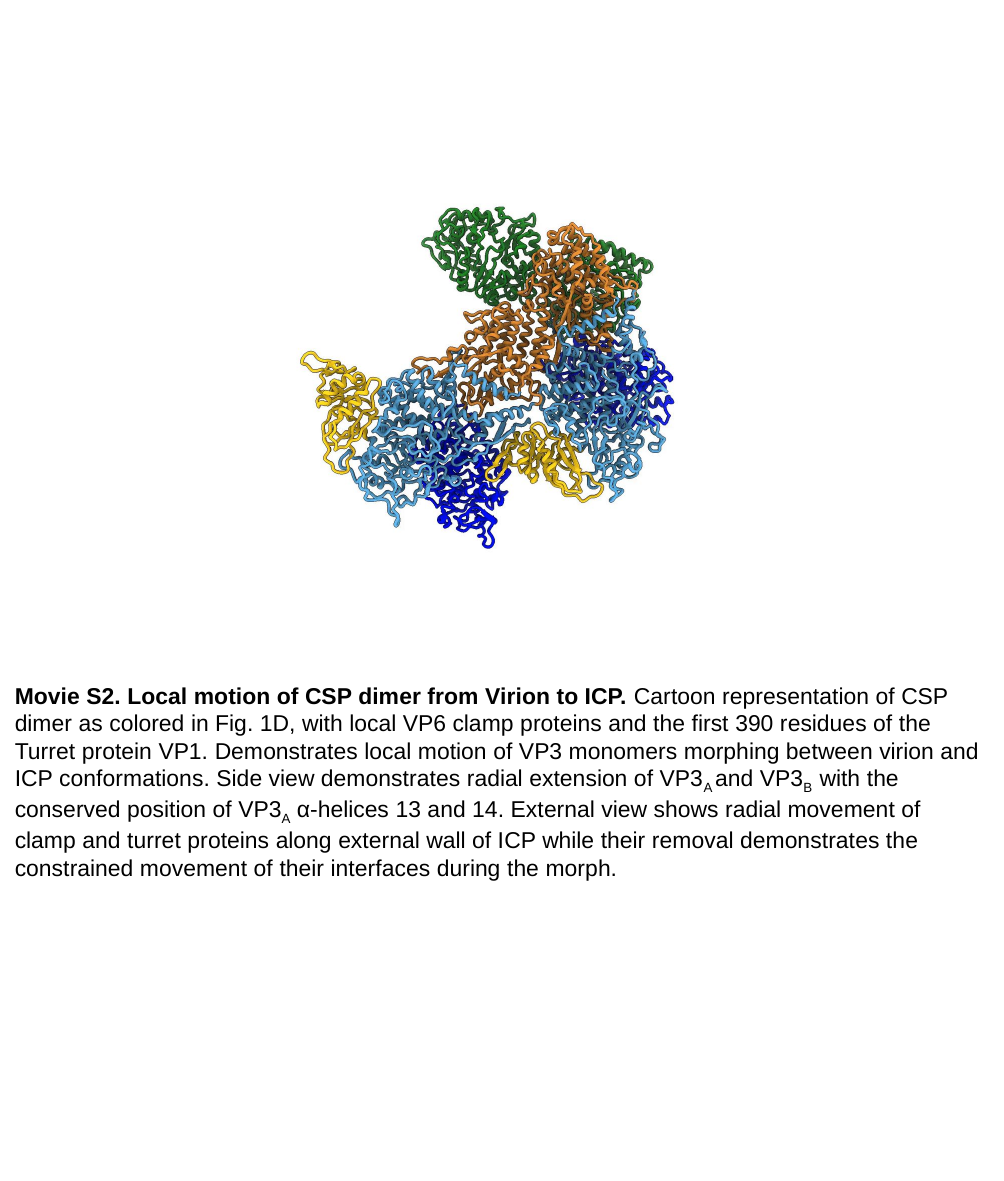

Movie S2. Local motion of CSP dimer from Virion to ICP. Cartoon representation of CSP dimer as colored in Fig. 1D, with local VP6 clamp proteins and the first 390 residues of the Turret protein VP1. Demonstrates local motion of VP3 monomers morphing between virion and ICP conformations. Side view demonstrates radial extension of VP3A and VP3B ­ with the conserved position of VP3A α-helices 13 and 14. External view shows radial movement of clamp and turret proteins along external wall of ICP while their removal demonstrates the constrained movement of their interfaces during the morph.

## Slide 3
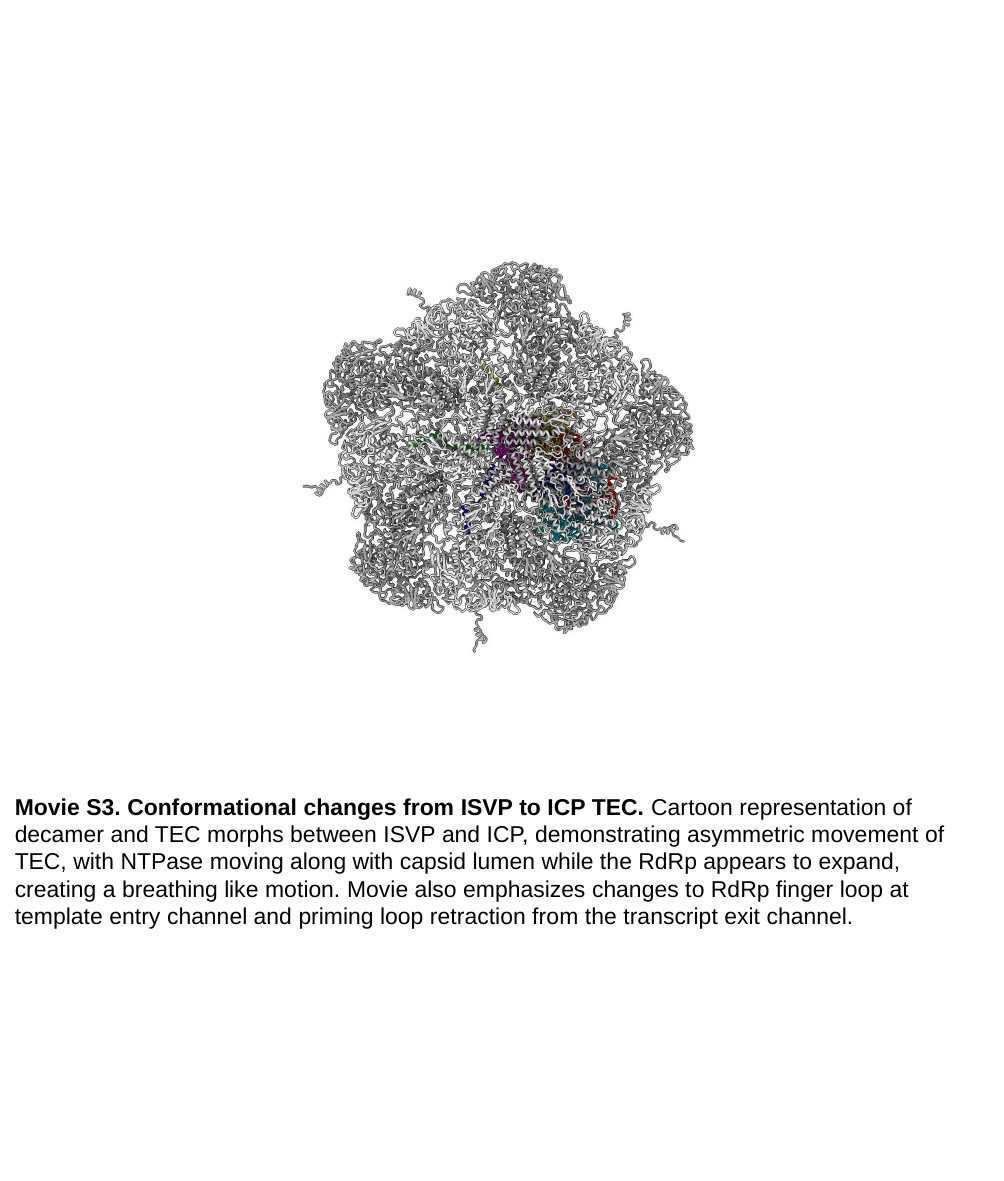

Movie S3. Conformational changes from ISVP to ICP TEC. Cartoon representation of decamer and TEC morphs between ISVP and ICP, demonstrating asymmetric movement of TEC, with NTPase moving along with capsid lumen while the RdRp appears to expand, creating a breathing like motion. Movie also emphasizes changes to RdRp finger loop at template entry channel and priming loop retraction from the transcript exit channel.

## Slide 4
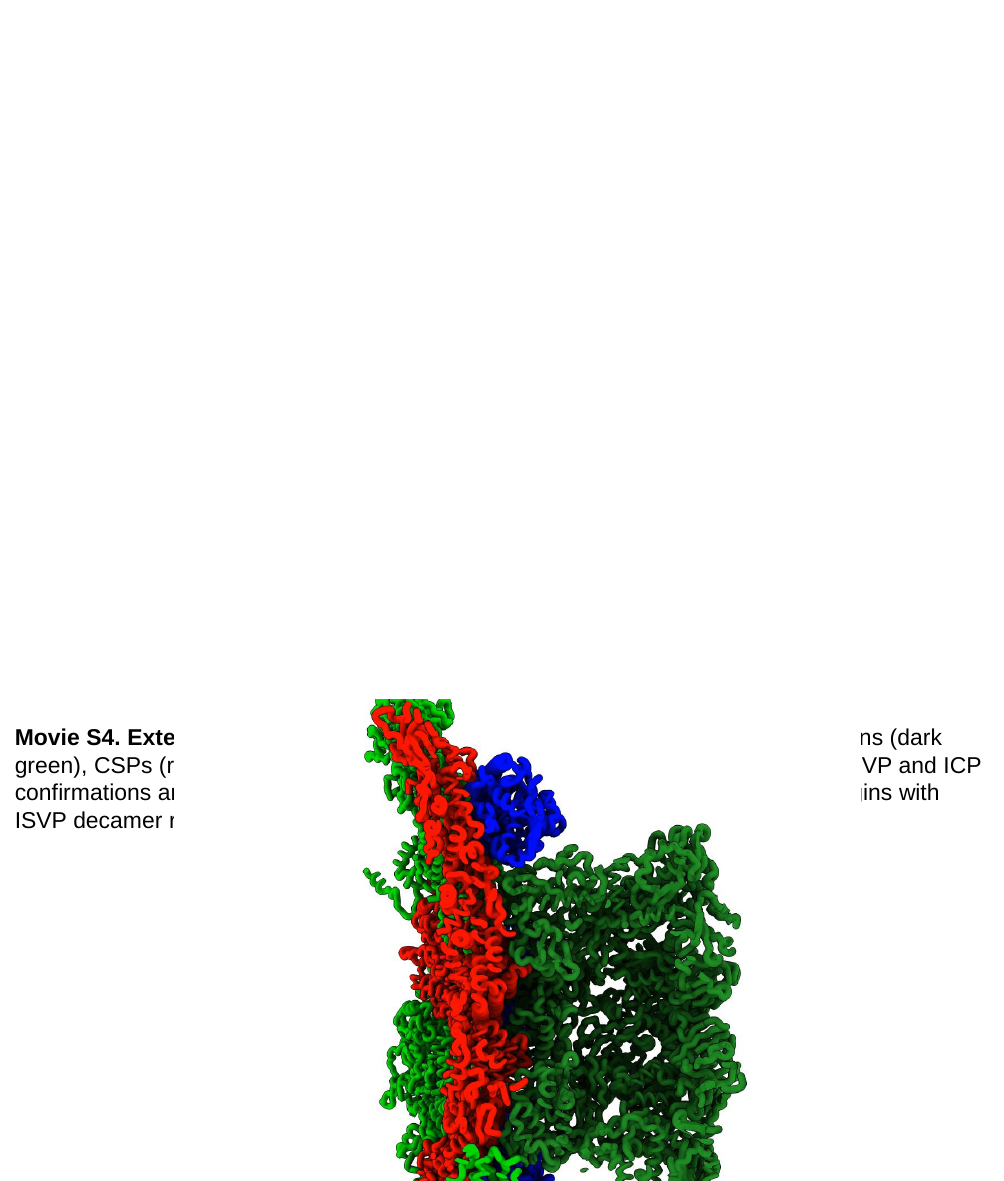

Movie S4. Extension of Turret proteins. Cross-sectional view of ARV turret proteins (dark green), CSPs (red and lime green), and clamp proteins (blue) morphing between ISVP and ICP confirmations and positions relative to the origin of the complete particle. Movie begins with ISVP decamer represented as cartoons morphing to ICP conformations.
